# BOTH HEALTH CARE PROFESSIONALS AND PATIENTS HARBOR MISPERCEPTIONS ABOUT DEMENTIA

**DOI:** 10.1093/geroni/igac059.213

**Published:** 2022-12-20

**Authors:** Laura Mehegan, David Parkes

**Affiliations:** AARP, Washington, District of Columbia, United States; AARP, Washington, District of Columbia, United States

## Abstract

Healthcare providers underestimate the willingness of adults to engage in a healthier lifestyle to potentially slow the progress of the disease and the willingness of patients to participate in research. Few adults recognize the impact lifestyle modifications have on the risk for cognitive decline and dementia, but some significant differences exists among perceptions amongst diverse communities. While most adults are willing to modify selected brain-healthy behaviors, relatively few currently engage in brain-healthy behaviors all or most of the time. Numerous discrepancies exist between the realities of dementia and overall feelings about a diagnosis. Among the more startling findings is 48% of adults believe they will likely have dementia — far more than will actually develop it. Health care providers substantially overestimate the worry that adults age 40 and older would feel if they had dementia. While one in five adults (19%) said they would feel ashamed or embarrassed if they had dementia, a staggering seven in 10 providers (69%) said their patients would feel ashamed or embarrassed. These negative perceptions by healthcare providers carry over into the interactions they have with patients when dealing with cognitive function. Nine in 10 adults age 40 and older (91%) want to be told of a dementia diagnosis, but only 78% of providers said they always tell patients the truth. There is a recognition by everyone that early diagnosis is beneficial, but most adults over 40 are not aware there are treatments available for dementia. More than half of adults do not know that dementia cannot be cured.

